# The Beat

**Published:** 2007-08

**Authors:** Erin E. Dooley

## Indian Subcontinent Gets Environmental Monitoring

Members of an Indo-U.S. workshop report in the 13 April 2007 issue of *Science* that to understand and slow global environmental change, there needs to be a global network of environmental data collection. Currently, much of Africa and the Indian subcontinent lacks such a network, although these areas have considerable influence on the regional and global environment. To help alleviate this information shortage, the Indian government is developing a new monitoring network known as INDOFLUX. This network will generate the needed baseline data from which to evaluate future environmental changes in a country where much of the population depends on natural resources and coastal integrity.

## Manufacturing Energy Use: Room for Improvement

The U.S. EPA has released a report that outlines energy use trends in 12 manufacturing sectors representing about 85% of total industrial energy use in the country. *Energy Trends in Selected Manufacturing Sectors* shows that industry has significant opportunities for improving its environmental performance through adopting energy-efficient and clean-energy technologies. The report also projects that with a “business-as-usual” scenario, total energy consumption of the sectors studied could increase by 20% over 2004 levels in the next 13 years, and that CO_2_ emissions could increase by 14%. The EPA further determined that use of renewable fuels in industry is now higher than in the residential, commercial, and transportation sectors, and is growing.

## California Rules on Formaldehyde

Citing studies on throat cancer, workplace asthma, and increased asthma and allergies in children, the California Air Resources Board has adopted new restrictions on formaldehyde that will cut by nearly 60% the amount of the chemical emitted into air from the resins and glues used to bond plywood, particleboard, and medium-density fiberboard. The total amount of formaldehyde emitted into California’s air each year will be reduced from the current 650 tons to 150 tons. The new rule will be phased in beginning in 2009, with full implementation in 2012. It will apply to all products sold, used, or made for sale in the state. Manufacturers will need to obtain third-party certification, maintain records, and label all wood or wood products to show compliance with the law.

## GINA Passes in House

The Genetic Information Nondiscrimination Act (GINA), first introduced in the House 12 years ago, was finally approved by that chamber in April 2007. GINA prohibits the wrongful use of genetic information in the making of hiring and health insurance decisions. Genetic factors are linked to 15% of all cancers and 10% of adult chronic diseases such as heart disease and diabetes. More than 200 organizations from different sectors have endorsed GINA, which had bipartisan support in the House with more than 200 cosponsors. The bill now goes to the Senate, which has passed it twice in recent years. President Bush has already voiced his support for the legislation.

## 2007 Goldman Environmental Prize

**Figure:**
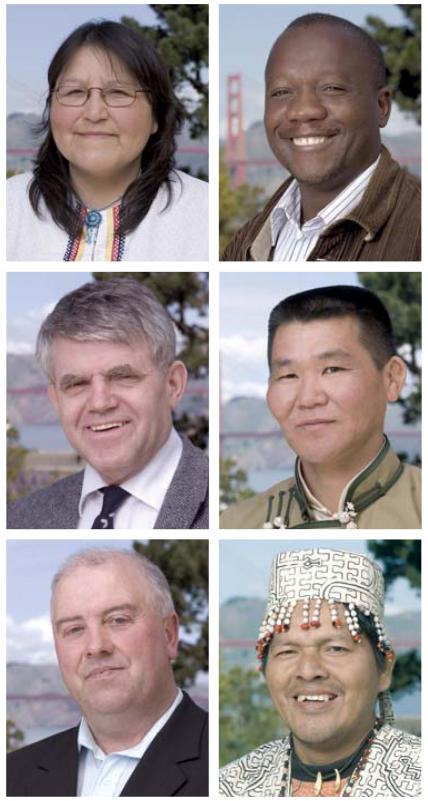


On 23 April 2007, the 2007 Goldman Environmental Prize was awarded in San Francisco to grassroots activists from around the world to honor their work in protecting the environment of their communities. This year’s winners are (above, clockwise from top left)

- Sophia Rabliauskas of Canada, who secured interim protection for the boreal forest of Manitoba against logging and hydropower development while the area’s future is decided by the government and international agencies.
- Hammerskjoeld Simwinga of Zambia, who developed an innovative sustainable community development program that protects biodiversity and helps alleviate poverty.
- Tsetsegee Munkhbayar of Mongolia, who worked with government and grassroots organizations to halt unregulated mining operations that threatened the country’s scarce water resources.
- Julio Cusurichi of Peru, who gained protection for an area of the remote Peruvian Amazon against logging and mining, preserving a sensitive ecosystem and the rights of indigenous peoples.
- Willie Corduff of Ireland, who headed a group of activists and landowners in successfully halting the construction of an illegally approved petroleum pipeline.
- Orri Vigfússuon of Iceland, who negotiated an agreement with governments and corporations to end destructive commercial salmon fishing practices in the region.

